# Tribomechanical Behaviour and Elasto-Plastic Contact Response of 3D-Printed Versus Conventional Polymer Inserts in Robotic Gripping Interfaces

**DOI:** 10.3390/polym18070891

**Published:** 2026-04-06

**Authors:** Georgiana Ionela Păduraru, Andrei Călin, Marilena Stoica, Delia Alexandra Prisecaru, Petre Lucian Seiciu

**Affiliations:** Department of Machine Elements and Tribology, National University of Science and Technology Politehnica, 060042 Bucharest, Romania; gipaduraru@upb.ro (G.I.P.); marilena.stoica@upb.ro (M.S.); delia.prisecaru@upb.ro (D.A.P.); lucian.seiciu@as.info.ro (P.L.S.)

**Keywords:** contact mechanics, elasto-plastic contact, tribomechanical response, scratch testing, anisotropy

## Abstract

Three-dimensional printed polymers produced using Fused Deposition Modelling (FDM) exhibit directional microstructures resulting from filament paths, layer interfaces, and cellular infill, leading to mechanical and tribological responses distinct from those of homogeneous bulk materials. This study presents a comparative tribomechanical evaluation of polypropylene (PP) bulk inserts and 3D-printed polyethylene terephthalate glycol (PETG) inserts with a 30% hexagonal infill, relevant for robotic gripping applications. Progressive scratch tests were performed under loads from 5 to 100 N (150 N for PP), and profilometry was applied to quantify groove morphology, ridge formation, and displaced-volume ratios. An elasto-plastic conical indentation model was used to derive indentation pressures and elastic–plastic transition radii from groove geometry. The PETG inserts exhibited heterogeneous groove depth, intermittent ridge tearing, and friction fluctuations associated with the internal infill structure, consistent with previous findings on anisotropy and architecture-dependent behaviour in additively manufactured polymers. In contrast, bulk PP demonstrated smoother friction profiles and more stable plastic flow under increasing loads. Two functional indices—specific frictional work and ridge-to-trace volumetric ratio—are introduced to support material selection for robotic gripping systems. The results show that local contact mechanics in 3D-printed inserts are governed by print-induced structural features and can be effectively evaluated through a scratch-based elasto-plastic analysis. The methods and results presented in this work support the rational selection and design of polymer inserts for robotic gripper fingertips. The proposed scratch-based elasto-plastic evaluation framework enables manufacturers and automation engineers to compare 3D-printed and conventional materials based on friction stability, wear response, and deformation resistance. This approach can be directly applied to optimise gripping performance in industrial handling, packaging, and collaborative robotics.

## 1. Introduction

Robotic systems have evolved rapidly over the last two decades, driven by accelerated progress in actuation technologies, sensing, and automated manipulation. Among these advancements, the performance of robotic hands and grippers has been central to enabling reliable interactions with fragile, irregular, or high-friction objects across industrial, medical, and research domains [1,2,3,4,5]. At the contact between the gripper and the object, frictional behaviour, plastic deformation, and surface damage strongly influence grasp stability, positioning accuracy, and long-term durability. Consequently, understanding how different materials respond to mechanical and tribological loading is essential for designing reliable robot–object interfaces.

Additive manufacturing, particularly Fused Deposition Modelling (FDM), introduces directional microstructures defined by filament orientation, interlayer bonding, and infill architecture, all of which govern anisotropy and architecture-dependent tribological behaviour, as widely reported in the literature [6,7,8,9]. Additive manufacturing has become a cornerstone in this area due to its ability to produce customised inserts with complex geometries at low costs and short lead times [10,11,12]. Numerous studies have demonstrated that printing parameters, such as infill density, layer thickness, and raster angle, directly influence surface quality, microstructural anisotropy, and wear resistance in printed polymers [11,12,13]. Portoacă et al. showed that modifying infill percentage and layer height has significant consequences for surface texture and wear behaviour in ABS and PLA inserts [10], while Gao and Wang highlighted the critical role of surface texture in improving the long-term durability of printed elements subjected to sliding loads [11]. Comparative studies between manufacturing processes further reinforce these microstructural effects; Hozdić found that SLA-printed samples display superior tensile strength and stiffness compared to FDM-printed ABS, due to differences in crosslinking, layer bonding, and microstructural uniformity [12].

Functional components subjected to cyclic or tangential loading—such as gears, cams, or gripper inserts—demonstrate strong dependence on wear resistance and surface stability. Studies on polymeric gears printed from ABS, PLA, and PETG reveal that infill density, raster direction, and print orientation significantly affect wear depth and service life [13]. These results emphasise that FDM materials cannot be treated as homogeneous solids. Instead, their mechanical and tribological performance reflects a combination of polymer properties and architectured structural features.

At the same time, a parallel body of research emphasises the fundamental role of contact mechanics in determining friction and wear behaviour. Elasto-plastic indentation models provide essential tools for predicting load distribution, local deformation, and subsurface stress in polymer surfaces [14,15]. Rough-surface contact behaviour, reviewed by Taylor, shows that friction and wear depend on multi-scale roughness, material stiffness, and lubrication conditions—factors especially relevant to dry contact in robotic gripping [16]. Advanced polymer formulations such as Tough PLA (TPLA) have been evaluated using definitive screening designs, highlighting the influence of layer height, infill density, and wall thickness on tensile, flexural, and compressive behaviour [17]. Meanwhile, carbon-reinforced PLA and PETG composites demonstrate substantial improvements in stiffness and friction coefficient reduction, linking microstructural reinforcement to tribological performance [18].

Material selection for gripper inserts must, therefore, balance mechanical strength, friction stability, wear resistance, and manufacturability. Polypropylene (PP) offers chemical resistance, low density, and smooth sliding behaviour, making it a widely used material for consumer and industrial components. Polyethylene terephthalate glycol (PETG) combines high impact strength, stability at elevated temperatures, and ease of processing, which has led to its increasing adoption in functional 3D-printed components. PETG’s resistance to cracking, toughness, and dimensional stability make it a strong candidate for contact surfaces exposed to repeated mechanical loading [13,19].

Given the structural anisotropy imparted by FDM and the importance of surface deformation in frictional contact, there is a need for a systematic tribomechanical evaluation of 3D-printed inserts compared to conventional bulk polymers. Scratch testing provides a controlled means of probing local elasto-plastic behaviour, capturing groove formation, ridge evolution, and frictional response under progressive loading. However, few studies directly link scratch morphology to underlying contact models or interpret print-induced heterogeneity in terms of composite-like deformation mechanisms.

This study addresses these gaps by examining the tribomechanical behaviour of bulk PP and 3D-printed PETG inserts produced with a 30% hexagonal infill. Progressive scratch tests were conducted under increasing normal loads, and surface profilometry was used to quantify groove depth, ridge geometry, and displaced-volume ratios. An elasto-plastic conical indentation model was used to convert surface profiles into mechanical descriptors such as indentation pressure and elastic–plastic separation radius. Finally, the study introduces application-oriented durability metrics—specific frictional work and ridge-to-trace volumetric ratio—relevant for selecting materials and infill configurations for robotic gripping.

While polypropylene (PP) and PETG differ in chemical composition and manufacturing route (bulk extrusion versus FDM printing), the comparison in this study is intentionally application-driven rather than chemistry-controlled. In robotic gripping systems, designers frequently face a practical choice between conventional homogeneous polymer sheets and architectured additively manufactured inserts that enable rapid customisation of geometry and compliance. Therefore, the objective of this work is not to isolate polymer chemistry effects under identical processing routes, but to evaluate how a widely used bulk industrial polymer (PP) performs relative to a structurally engineered FDM insert (PETG with controlled infill) under identical contact conditions. This functional comparison reflects real-world material selection scenarios in automation and handling systems, where performance is governed by the combined influence of polymer properties and structural architecture. In the present analysis, particular attention is given to identifying deformation patterns and frictional fluctuations that correlate with infill geometry rather than intrinsic material chemistry. Periodic COF modulation, heterogeneous groove morphology, and load-dependent ridge instability are interpreted as architecture-driven effects, whereas smooth monotonic responses are associated with homogeneous bulk behaviour. This distinction allows the respective roles of material chemistry and manufacturing architecture to be qualitatively separated.

PETG was selected as the representative FDM material due to its widespread industrial adoption for functional load-bearing printed components, superior interlayer bonding compared to printed polypropylene, and its established dimensional stability in dry sliding applications.

Through this combined experimental–theoretical framework, the work provides new insights into how print-induced microstructures shape contact mechanics in 3D-printed polymers and offers practical guidance for designing durable, high-performance inserts for robotic manipulation.

## 2. Materials and Methods

### 2.1. Elasto-Plastic Contact Framework for Conical Indentation

**Figure 1:**
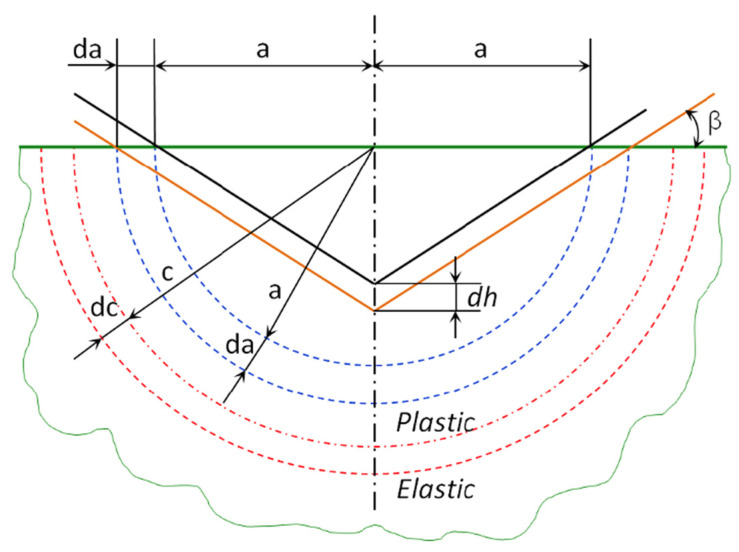
Model for elasto-plastic indentation.

The tribomechanical response of the tested materials was interpreted using an elasto-plastic indentation model originally described by Johnson [20]. The model assumes that deformation under a rigid indenter is approximately radial and can be idealised as a hemispherical plastic zone of radius *c* surrounded by an elastic field, as seen in Figure 1. When a rigid cone penetrates the surface, the plastically deformed region is represented as a hemispherical cavity subjected to a uniform hydrostatic pressure *p*_0_, while stresses outside this cavity decay radially.

Inside the plastic zone (*r* < *c*), the radial and tangential normal stresses are expressed as:

(1) $$\sigma_{r}=\;-k$$

(2) $$\sigma_{t}=-k$$

where:

- σ_r_ = radial normal stress;
- σ_t_ = tangential (hoop) normal stress;
- k = flow stress (shear yield stress);
- The minus sign “−” indicates compressive stress.

The flow stress, k, represents the material’s resistance to plastic (permanent) deformation inside the plastic zone created by the indenter.

More specifically, in the classic Johnson (1985) elasto-plastic indentation model, k is the equivalent shear yield stress of the material [20]. It is directly related to the uniaxial yield strength *σ_y_* by:

(3) $$k=\frac{\sigma_{y}}{\sqrt{3}}$$

This comes from the von Mises yield criterion, which governs plastic deformation in isotropic materials. Thus, once yielding begins, the material within the plastic region supports a constant hydrostatic compressive stress. Inside the plastically deformed region beneath the indenter (*r* < *c*), the following assumptions can be made:

- The stress state is static compression;
- The material flows plastically;
- The radial and hoop stresses are equal and constant.

(4) $$\sigma_{r}=\sigma_{t}=-k$$

This implies that:

- Plastic flow begins when the static pressure reaches k;
- k controls the onset and magnitude of plastic zone expansion;
- Higher k leads to a deeper elastic–plastic transition radius, which, in turn, leads to a narrower groove and ridge.

### 2.2. Rigid Cone–Plastic Half-Space Model

**Figure 2:**
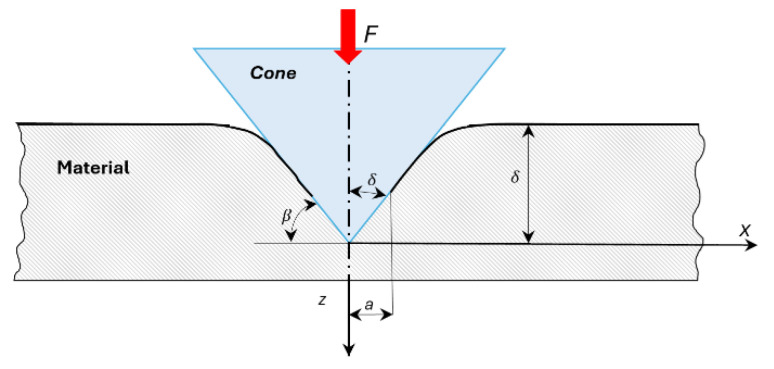
Cone-plane contact geometry.

To complement the elasto-plastic cavity model, the indentation response was also interpreted using the rigid cone–plastic half-space formulation. Figure 2 below shows the basic geometry of the contact between a rigid cone and a plastic half-space.

For a rigid conical indenter of semi-angle *θ* penetrating to a depth *h*, the resulting surface profile is:

(5) z(r)=rcotθ

The indentation problem can be expressed through the axisymmetric Abel-type integral equation:

(6) $$u(r)=\frac{2}{\pi E^{*}}\int_{0}^{a}\frac{p(t)\;t\;dt}{t+r}$$

where:

- u(r) is the radial surface displacement;
- p(t) is the distributed contact pressure;
- $E^{*}$ is the plane-strain modulus;
- *a* is the contact radius.

Assuming perfectly plastic behaviour with a constant yield stress *k*, the applied load reduces to:

(7) $$F=\frac{2k}{3\tan\theta }\;$$

Accordingly, the mean contact pressure becomes:

(8) $$p_{m}=\frac{2k}{3\tan\theta }$$

which implies that, for an ideally plastic material, the average pressure beneath the indenter is independent of penetration depth and depends solely on the cone geometry and the material’s flow stress.

The elastic energy stored during indentation follows:

(9) $$U=\frac{Fh}{2}$$

This analytical framework was used as a consistency check for the experimentally measured groove depths and contact pressures derived from profilometry.

### 2.3. Determination of Surface Deformation by Progressive Scratch Testing

**Figure 3:**
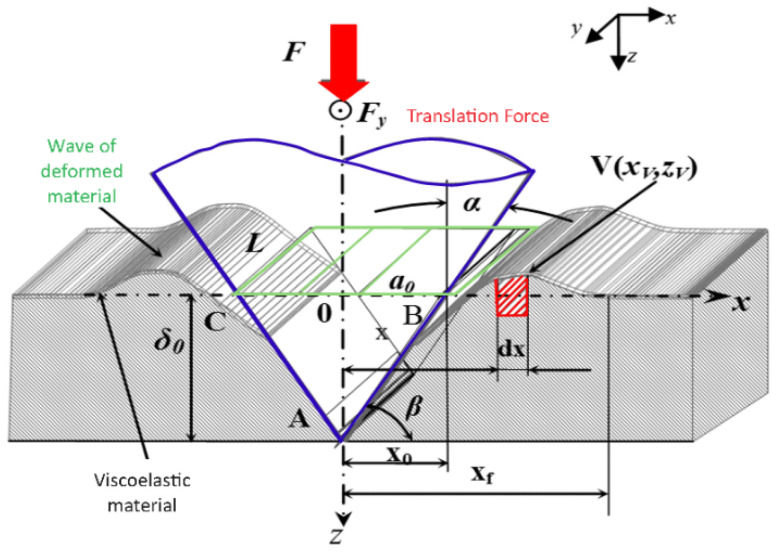
Conical contact through scratching.

Progressive-load scratch testing was used to quantify the plastic deformation behaviour of the materials. As the conical indenter traverses the surface under gradually increasing loads, it generates a groove accompanied by ridge formation on either side due to displaced material. Figure 3 below illustrates the principle of scratching.

**Figure 4:**
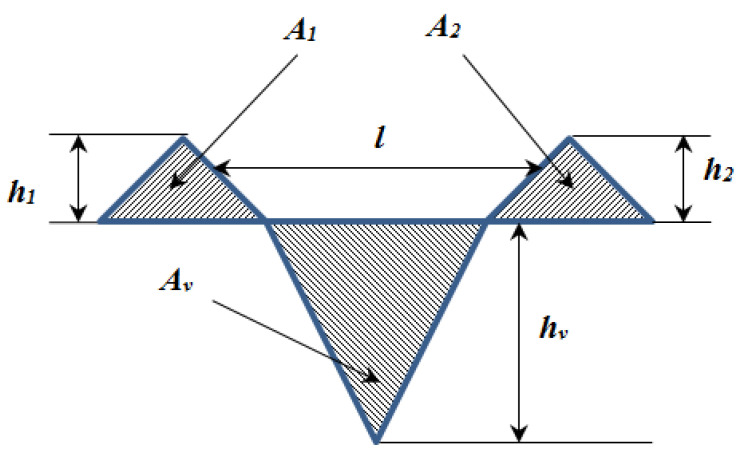
Representation of the volume of material displaced during the wear process and the wear trace volume.

Assuming a parabolic form of the ridge cross-section, as depicted in Figure 4 below, the volume balance between the material removed from the groove and that displaced into the ridges is:

(10) $$V_{ridge}=V_{groove}$$

The ridge-to-trace volumetric ratio, used as a work-hardening indicator, is defined as:

(11) $$\gamma =\frac{A_{ridge}}{A_{groove}}$$

where:

- $A_{groove}$ is the groove cross-sectional area;
- $A_{ridge}$ is the combined area of the two ridges.

Values of γ>1 denote significant plastic accommodation and ridge build-up, while γ<1 indicates material removal or local fracture.

(12) $$A_{1}+A_{2}=A_{v}$$

Using these relationships, the ridge geometry, groove depth, and displaced volume were extracted from profilometry scans and linked to the underlying elasto-plastic behaviour of PP and PETG.

### 2.4. Experimental Setup

**Figure 5:**
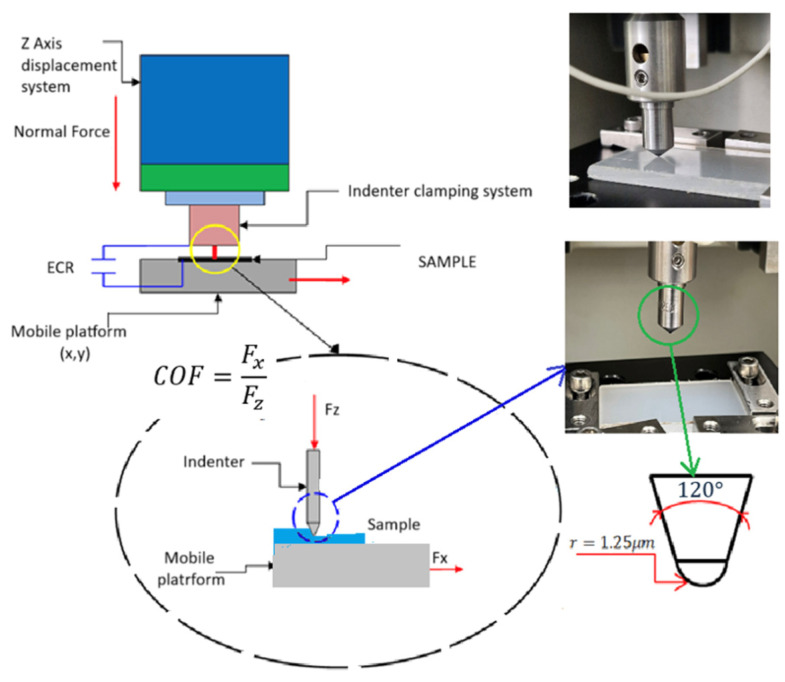
Operational scheme of the CETR UMT Multi-Specimen Test System.

Scratch tests were carried out using a CETR UMT Multi-Specimen Test System (Center for Tribology, Inc., Campbell, CA, USA) equipped with a diamond-coated conical stylus (cone angle 60°, tip radius 1.25 µm) depicted in Figure 5 (below). Each scratch was conducted over a 10 mm distance at a constant sliding speed of 0.5 mm/s for a total duration of 20 s. The applied normal load increased linearly along the scratch path.

The loading schedule was:

- 5, 10, 20, 50, 100 N for all samples;
- 150 N was applied additionally for polypropylene (PP) samples.

The instrument recorded:

- Normal force ($F_{z}$);
- Tangential force ($F_{x}$);
- Real-time coefficient of friction (COF).

The samples consisted of two bulk PP specimens (PP1 and PP2) and a 3D-printed PETG sample with a 30% hexagonal infill. All specimens were machined to 45 mm × 30 mm × 3 mm and cleaned with isopropyl alcohol prior to testing. Each sample was rigidly clamped to eliminate vibration and ensure repeatability. For each material, one progressive-load scratch was performed for each target maximum load along separate, non-overlapping tracks to avoid interference between deformation zones. The continuous load increase along the 10 mm scratch length enabled sampling of multiple local microstructural regions, particularly in the case of the architectured PETG specimen. The two polypropylene specimens (PP1 and PP2) were not treated as statistical replicates for averaging purposes, but as representative samples used to verify the consistency of bulk material behaviour under slightly different initial surface conditions typical of industrial extruded sheets. The objective of the study is not to perform statistical analysis, but to enable a mechanism-oriented comparison between a homogeneous material (PP) and an architectured material (3D-printed PETG with controlled infill).

The bulk polypropylene specimens (PP1 and PP2) were machined from Polystone^®^, an extruded polypropylene sheet manufactured by Röchling Engineering Plastics SE & Co. KG (Haren, Germany).

According to the manufacturer’s technical datasheet, the material corresponds to an isotactic polypropylene (PP) grade with the following nominal properties:

- Density: 0.95 g/cm^3^;
- Tensile yield strength: 22 MPa;
- Tensile modulus: 800 MPa;
- Elongation at break: >50%;
- Charpy impact strength (V-notch): 12 kJ/m^2^;
- Shore D hardness: 63;
- Melting temperature: 135 °C;
- Vicat softening temperature (B): 67 °C.

The material exhibits low water absorption (<0.01%) and good chemical resistance, typical of general-purpose engineering polypropylene sheets used in industrial applications. Sheets were supplied in plate form and machined to the final specimen dimensions (45 mm × 30 mm × 3 mm) prior to testing.

The additively manufactured specimens were produced from AzureFilm PETG Original Black (1.75 mm diameter) filament supplied by AzureFilm (Sežana, Slovenia). The filament is categorised as a glycol-modified PET co-polyester (PETG), widely used in functional FDM printing due to its combination of ductility, durability, and ease of processing.

According to the manufacturer’s technical data and typical supplier datasheets for AzureFilm PETG, the material exhibits the following representative characteristics:

- Diameter tolerance: ±0.02 mm.
- Recommended printing temperature: 220–240 °C, and heated bed temperature: 80–90 °C.
- Tensile yield strength: ~51 MPa (ISO 527-2).
- Tensile modulus: ~2980 MPa (ISO 527-2).
- Elongation at break: ~29.

These properties correspond to typical engineering PETG filament grades used in mechanically functional 3D-printed parts and provide a sound basis for comparing load-dependent scratch behaviour with the bulk PP reference.

All PETG specimens were fabricated using a Flashforge Creator Pro FDM printer (Zhejiang Flashforge 3D Technology Co., Ltd., Hangzhou, China) operating in single-extruder mode.

The printing parameters were as follows:

- Nozzle diameter: 0.4 mm;
- Layer height: 0.2 mm;
- Raster angle: ±45° alternating between successive layers;
- Extrusion temperature: 235 °C;
- Build plate temperature: 80 °C;
- Infill pattern: hexagonal (honeycomb);
- Infill density: 30%;
- Perimeters (shells): 3;
- Top/bottom solid layers: 4.

These parameters were selected to ensure stable printing, good interlayer bonding, and a representative internal architecture for functional PETG components.

Both PP and PETG samples were prepared with nominal dimensions of 45 mm × 30 mm × 3 mm. PP samples were machined from bulk sheet material to final dimensions. The PETG samples were tested in the as-printed condition, without post-processing or surface machining, in order to preserve the native print-induced surface morphology relevant for robotic gripping applications. Prior to testing, all samples were cleaned using isopropyl alcohol (IPA) to remove dust, grease, and loose debris.

Initial surface roughness was measured before scratch testing using a Mahr GD 140 contact profilometer.

Representative average roughness values were:

PETG (as-printed):

- Ra = 7.5 ± 1.2 µm;
- Rz = 50 ± 10 µm.

PP (machined bulk):

- Ra = 1.4 ± 0.3 µm;
- Rz = 11 ± 3 µm.

These values reflect the inherent differences between as-printed FDM surfaces and machined bulk polymers, and were taken into account when interpreting scratch-induced deformation and frictional response.

### 2.5. Surface Profilometry and Data Extraction

**Figure 6:**
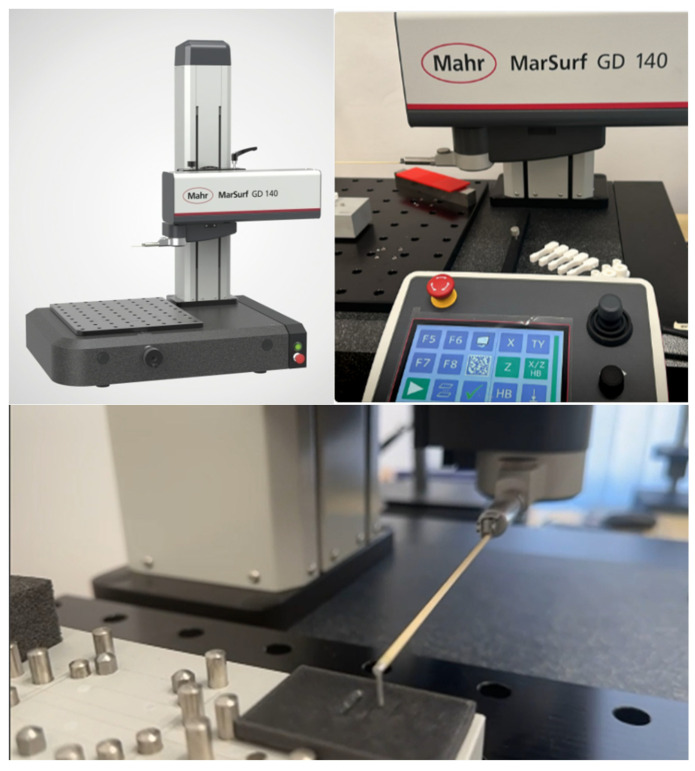
Mahr GD 140 roughness measuring station.

Post-test surface characterisation was performed using a Mahr MahrSurf GD 140, Göttingen, Germany, contact profilometer (Figure 6) capable of both 2D line profiles and 3D surface topography.

For each scratch, the following parameters were extracted:

- Groove depth and width;
- Ridge height and symmetry;
- Displaced material volume;
- Surface roughness and waviness (Ra, Rz, and Wt);
- Cross-sectional geometry of groove and ridges;
- Local slope and curvature.

These measured profiles were used to compute:

- Elastic–plastic separation radius *c*;
- Indentation pressure *p*_0_;
- Ridge-to-groove volumetric ratios γ;
- Specific frictional work per unit scratch length.

## 3. Results

**Figure 7:**
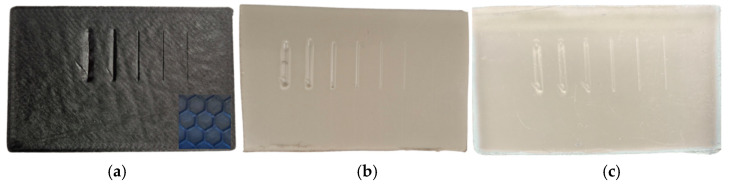
Test samples: (**a**)—PETG (30% hexagonal infill), (**b**)—PP1, and (**c**)—PP2.

Three samples were tested: one 3D-printed PETG specimen with a 30% hexagonal infill and two polypropylene specimens (PP1—white, PP2—semi-transparent), all with dimensions 45 × 30 × 3 mm. Five progressive-load scratches were performed on each sample at 5, 10, 20, 50, and 100 N; PP samples were additionally tested at 150 N due to their higher load-bearing capacity. The coefficient of friction (COF), groove morphology, ridge formation, and displaced-volume metrics were extracted from scratch signals and profilometry data(Figure 7).

### 3.1. Coefficient of Friction (COF) Evolution Under Progressive Loading

**Figure 8:**
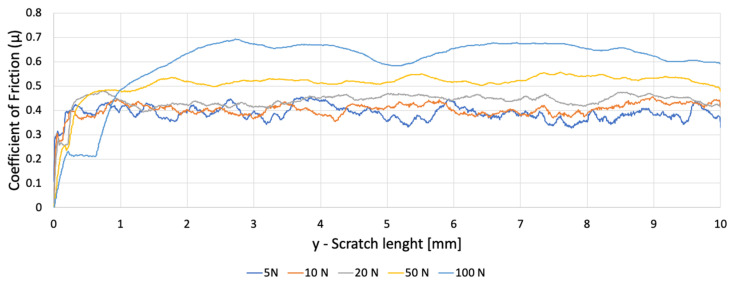
Variation of the coefficient of friction as a function of scratch length for PETG.

The PETG sample exhibited strong frictional variability along the scratch track (Figure 8).

Fluctuations increased with load and corresponded to:

- Transitions between solid filament regions and infill voids;
- Rupture of inter-bead bridges;
- Localised stiffening occurred when the indenter encountered rib intersections.

The friction traces showed periodic modulations whose spacing matched the hexagonal infill cell size, confirming architecture-dependent contact behaviour. PETG consistently exhibited higher COF dispersion, reflecting its composite-like, nonhomogeneous internal structure.

**Figure 9:**
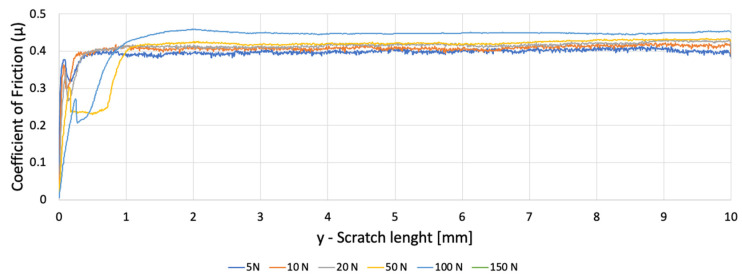
Variation of the coefficient of friction as a function of scratch length for PP_1_.

**Figure 10:**
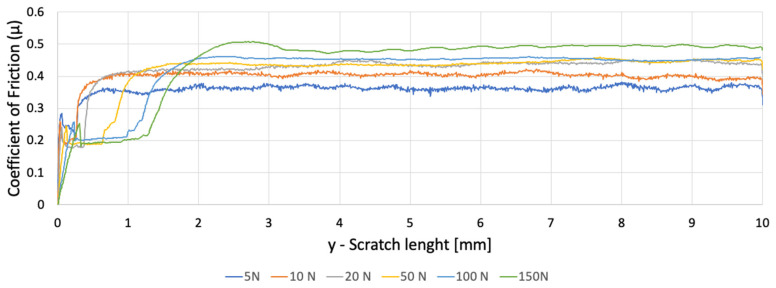
Variation of the coefficient of friction as a function of scratch length for PP_2_.

Both PP samples generated smoother, more stable COF curves (Figure 9 and Figure 10). Variations along the track were minor and increased only slightly at higher loads. This behaviour is characteristic of homogeneous, ductile ploughing, with minimal influence from microstructural discontinuities. Differences between PP1 and PP2 were limited to small offsets in absolute COF magnitude, attributable to surface finish and initial roughness. The close agreement between PP1 and PP2 confirms that the observed response is characteristic of homogeneous polypropylene and not dependent on specimen-specific variability.

**Figure 11:**
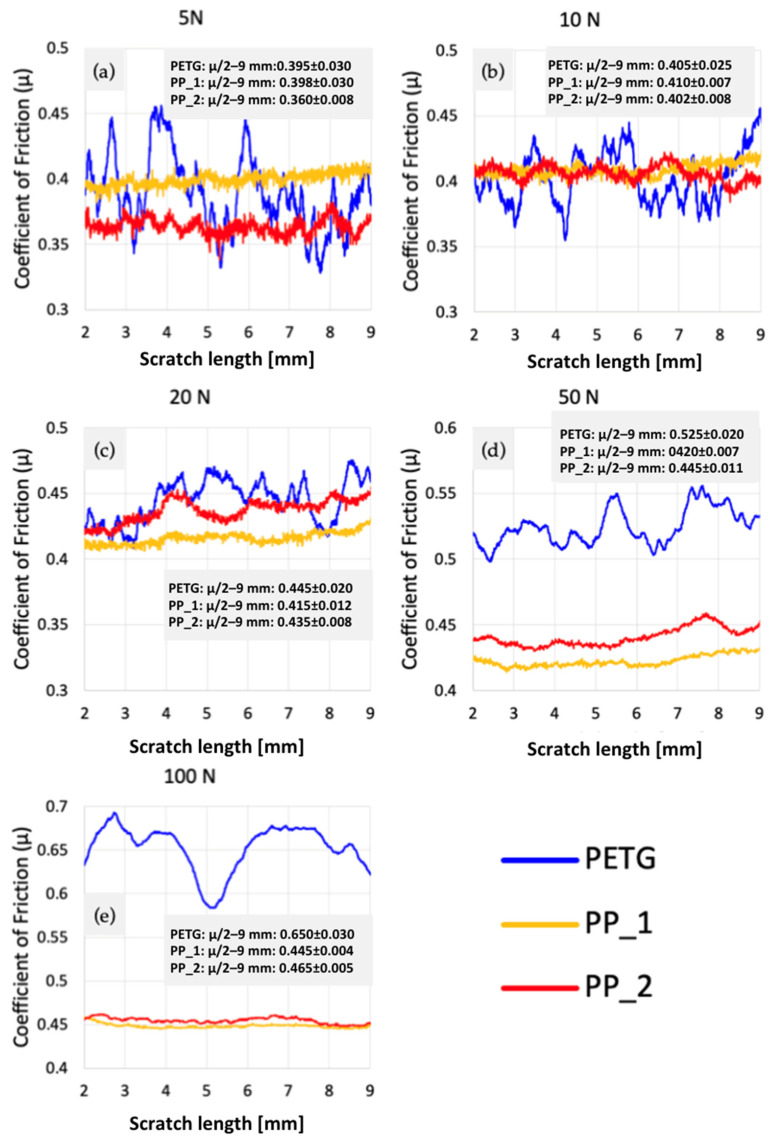
Variation of the coefficient of friction as a function of scratch length for all the test samples at (**a**) 5 N, (**b**) 10 N, (**c**) 20 N, (**d**) 50 N, and (**e**) 100 N.

Figure 11 shows that at matched loads:

- PETG exhibits the highest COF variability;
- PP1 and PP2 exhibit stable COF evolution;
- At 5–20 N, PETG already shows micro-instabilities not present in PP.

These findings indicate that frictional behaviour in PETG is dominated by infill-driven stiffness modulation, while PP responds uniformly as expected for a bulk polymer.

### 3.2. Groove Depth, Width, and Scratch Morphology

**Figure 12:**
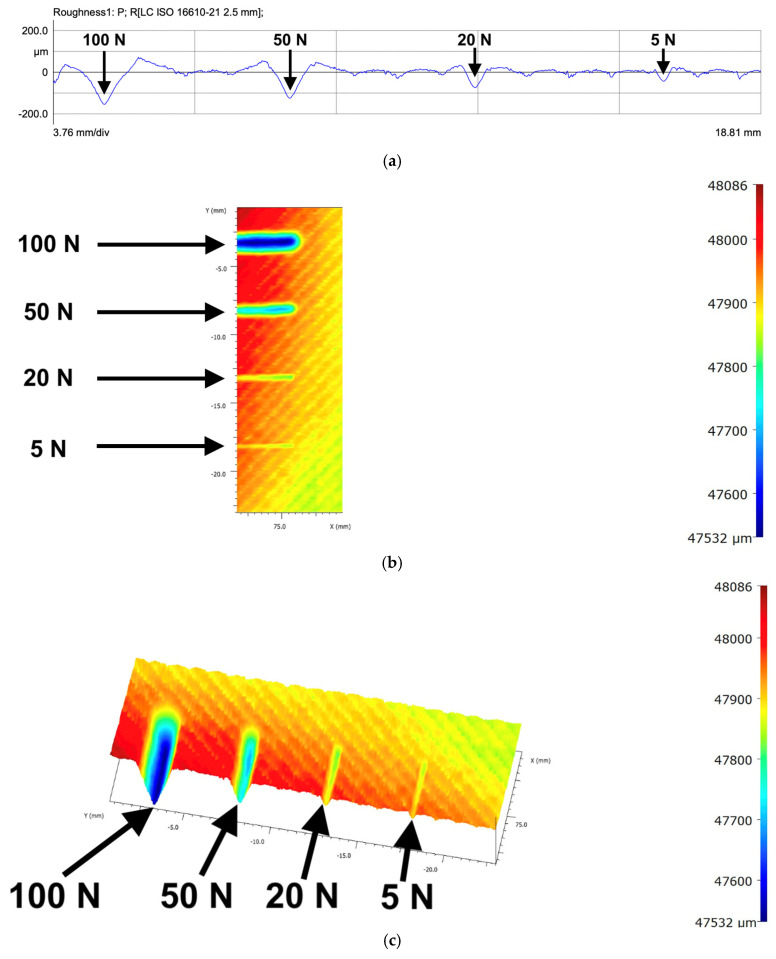
Profilometry images for the PETG sample: (**a**) roughness profile, (**b**) 2D height map, and (**c**) 3D heat map.

Profilometry revealed heterogeneous groove depths, periodic widening, and asymmetric profiles (Figure 12). These features correlate with:

- Local densification when the conical indenter crossed filament intersections;
- Sudden penetration when entering low-density infill regions;
- Ridge tearing produced by brittle ligament separation.

Figure 12a presents a linear roughness profile acquired over an 18.81 mm scan length of the PETG surface. Four distinct groove signatures are visible, corresponding to the applied loads of 100 N, 50 N, 20 N, and 5 N (from left to right). The profile exhibits pronounced peaks and valleys within a vertical range of approximately −200 to +200 μm, reflecting load-dependent material displacement and surface damage.

Figure 12b shows the corresponding 2D height map, where the colour scale represents local surface elevation. Lower regions (blue) indicate deeper penetration of the stylus, while higher ridges and undisturbed areas appear in red and yellow. The spatial periodicity of the infill structure is weakly visible in the pattern of alternating elevations.

Figure 12c provides a 3D topographic reconstruction of the same region. The depth scale reveals maximum groove depths approaching ~550 μm under the highest load. The three-dimensional view highlights the asymmetry and non-uniformity of ridge formation, as well as the progressively increasing deformation with higher applied forces.

**Figure 13:**
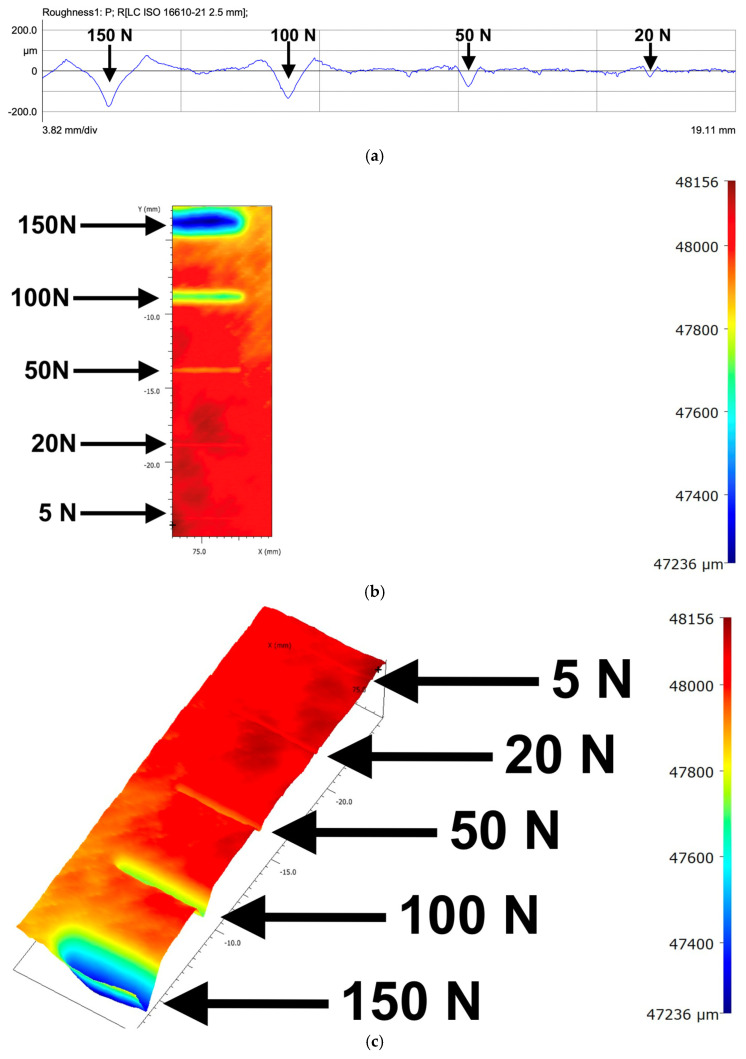
Profilometry images for the PP_1_ sample: (**a**) roughness profile, (**b**) 2D height map, and (**c**) 3D heat map.

**Figure 14:**
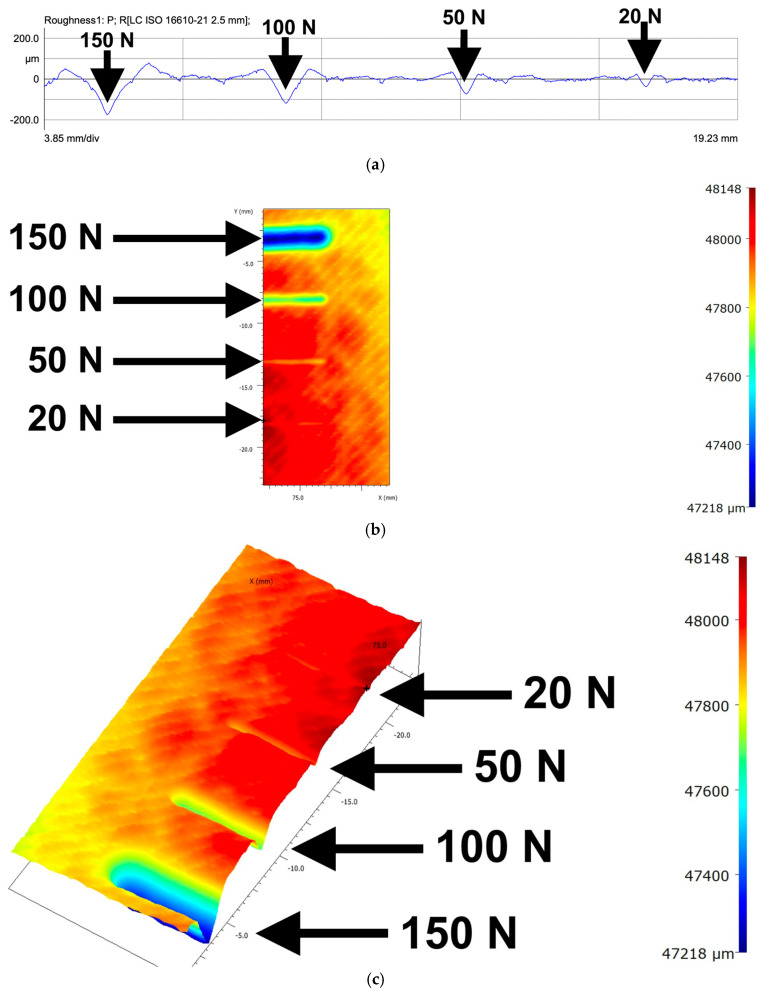
Profilometry images for the PP_2_ sample: (**a**) roughness profile, (**b**) 2D height map, and (**c**) 3D heat map.

PP specimens displayed deeper and more uniform grooves (Figure 13 and Figure 14). PP2 produced the deepest grooves (≈930 μm), but both PP samples exhibited:

- Symmetric ridge formation;
- Continuous ploughing behaviour;
- Absence of periodic discontinuities.

These smooth profiles reflect homogeneous yielding and efficient plastic accommodation under increasing normal loads.

At 5 N, the PP samples displayed very shallow or nearly imperceptible grooves, demonstrating strong resistance to initial indentation. PETG, in contrast, presented visible profiles even at low loads due to its lower local stiffness in infill regions.

For both PP1 and PP2, the absence of a visible scratch at the lowest applied force demonstrates their high resistance to initial penetration and low-load damage. This response reflects the ductile and homogeneous nature of bulk polypropylene, which accommodates small stresses elastically before transitioning into plastic flow. By contrast, PETG—particularly in its 3D-printed configuration—exhibits noticeable surface marking even under low normal loads. This behaviour arises from its architectured internal structure, where infill porosity, bead interfaces, and local stiffness variations reduce its resistance to early-stage indentation.

### 3.3. Quantitative Indices: Ridge-to-Trace Ratio and Specific Frictional Work

The ridge-to-trace volumetric ratio (γ) and the specific frictional work per unit scratch length ($w_{f}$) were calculated from profilometry and tangential force data for all tested load levels. Table 1 summarises the mean values obtained for PETG, PP1, and PP2.

PETG exhibits load-dependent γ behaviour, with γ < 1 at 5 N and 100 N, indicating limited plastic accommodation and partial material removal under low and high load conditions. At intermediate loads (20–50 N), γ > 1 suggests dominant ploughing with ridge build-up.

PP samples show a more stable γ evolution, although transitions between ploughing-dominated and removal-dominated regimes are also observed at specific loads.

## 4. Discussion

The experimental results show that the tribomechanical response of 3D-printed PETG and bulk PP inserts is governed by the way plastic flow develops beneath the conical indenter and by the degree of heterogeneity intrinsic to each material system. Bulk PP, being compositionally and structurally uniform, generated smooth and largely monotonic coefficient-of-friction (COF) profiles across all loads, accompanied by continuous groove growth and symmetric ridge formation. This behaviour reflects a predominantly ductile ploughing mechanism, with limited micro-cutting and efficient material accommodation in the ridge regions. The stability of PP under progressive loading is further evidenced by its consistent ridge/groove morphology and the absence of periodic discontinuities in both 2D and 3D profilometry maps. The consistency observed between the two PP specimens further supports that the bulk response is governed by material homogeneity, whereas the variability observed in PETG arises from its internal architecture rather than experimental scatter.

In contrast, PETG with a 30% hexagonal infill exhibited marked frictional variability and heterogeneous scratch morphology. The COF traces displayed periodic oscillations that aligned with the spacing of the cellular infill architecture, confirming that internal print geometry acts as a local stiffness modulator. Profilometry maps revealed alternating deep and shallow regions along the scratch path, ridge tearing, and non-uniform ridge build-up, all of which correspond to transitions between bead intersections and void-rich regions. These observations support the interpretation that 3D-printed PETG behaves as an architectured composite; its tribomechanical performance is dictated not only by bulk polymer properties (modulus, yield strength), but also by mesoscale structural constraints imposed by the infill topology.

By linking profilometry outputs to the elasto-plastic indentation model, the groove–ridge geometry could be translated into mechanical descriptors such as the indentation pressure and the elastic–plastic transition radius. This approach moves beyond qualitative observation and permits a field-level interpretation of the surface deformation. PETG’s fluctuating indentation parameters reflect the spatial variation in local stiffness and constraint, whereas the smooth parameter evolution observed in PP indicates a uniform subsurface stress field. These distinctions are critical for durability-by-design principles in robotic gripping, where consistent load transfer and predictable deformation are necessary for long-term reliability.

Reducing the experimental data to task-level performance indicators further clarifies material selection strategies for robotic gripping applications. The specific frictional work per unit scratch length captures the cumulative tangential energy dissipated up to a target penetration depth. Lower values—observed for PP—imply reduced thermal loading and are beneficial for continuous or repetitive handling tasks. The ridge-to-trace volumetric ratio (γ) provides a direct measure of plastic accommodation efficiency. PP exhibited γ > 1 across almost all tested loads, indicating dominant ploughing behaviour and well-developed ridge formation with γ below unity, suggesting partial material removal or incomplete ridge build-up under specific contact conditions. PETG, in contrast, displayed fluctuating γ values including γ ≤ 1 at some locations, signifying micro-fragmentation and reduced capacity for stable plastic accommodation.

The combined interpretation of COF stability, frictional work, and γ-ratio, therefore, supports a practical mapping between material type and robotic duty cycle. Bulk PP is suited for endurance and low-heat applications where predictable friction is essential, whereas PETG may be appropriate for tasks requiring high-surface compliance or controlled deformation, provided that the architectural heterogeneity is acceptable within the targeted operating window.

Wear mechanisms extracted from profilometry reinforce these behavioural trends. PP exhibited micro-ploughing with compact, lamellar debris and stable ridge formation, even as loads increased. PETG demonstrated alternating wear modes—ploughing, interfacial opening at bead boundaries, micro-cutting, and ridge tearing—consistent with transitional loading across infill interfaces. Ribbon-like debris at higher loads indicated intermittent brittle responses in the PETG structure. These features correspond directly to the load-dependent intermittency observed in the COF traces and to the increased dispersion in specific wear rates.

Overall, the mechanistic distinctions between the materials can be summarised as follows:

- PP: homogeneous plastic ploughing, limited cutting, stable ridge formation, low debris liberation, and smooth friction evolution.
- PETG: architecture-modulated ploughing, interfacial opening between beads, intermittent micro-cutting, ridge tearing, and elevated frictional variability.

These findings demonstrate that 3D-printed inserts cannot be assessed solely based on their nominal polymer identity; internal architecture must be explicitly considered to ensure predictable tribomechanical performance in robotic gripping systems.

## 5. Conclusions

This study provides a comparative tribomechanical assessment of bulk polypropylene (PP) and 3D-printed polyethylene terephthalate glycol (PETG) inserts with a 30% hexagonal infill, focusing on their suitability as contact elements in robotic gripping interfaces. The results show that PP exhibits high resistance to low-load scratching, stable frictional response, uniform plastic deformation, and efficient ridge formation. These characteristics are indicative of a homogeneous ductile substrate and make PP particularly suitable for applications requiring durability, repeatable contact behaviour, and low frictional heating during long-duty manipulation.

By contrast, PETG displayed visible indentation even at low loads and showed strong architecture-dependent effects arising from its infill geometry. Friction traces exhibited periodic fluctuations, and profilometry revealed heterogeneous groove morphology, ridge tearing, and infill-induced discontinuities. Although PETG demonstrated lower mean friction in some regimes, its higher frictional variability and heterogeneous wear mechanisms highlight the importance of explicitly considering infill topology in the design of robust 3D-printed gripping components.

Three key scientific questions were addressed:

(i) How infill-driven heterogeneity influences friction evolution, groove morphology, and ridge formation across increasing loads;
(ii) How groove–ridge geometry can be mapped to elasto-plastic contact fields relevant to durability and wear;
(iii) How task-level metrics—specific frictional work and ridge-to-trace volumetric ratio—enable rational material selection for distinct robotic gripping duties.

The proposed experimental–theoretical framework provides a direct, interpretable methodology for connecting scratch-derived deformation modes to functional performance in robotic gripping systems. The findings emphasise that print architecture significantly modifies local stiffness, stress distribution, and wear behaviour, underscoring the need for structured print-parameter selection when designing next-generation polymer inserts for automation.

Future work should extend the present analysis to additional infill geometries, layer heights, printing orientations, and environmental conditions such as temperature, humidity, and lubrication. Such studies will improve the generality of the proposed framework and support the development of architecture-aware design strategies for additively manufactured tribological components.

## Figures and Tables

**Table 1:** Ridge-to-trace ratio (γ) and specific frictional work ($w_{f}$).

Material	Load (N)	γ (–)	(w_f_) (J/m)
PETG	5	0.7	0.97
	10	1.44	2.02
	20	1.42	4.39
	50	1.42	12.77
	100	0.36	30.16
PP1	5	6.4	0.99
	10	3	2.03
	20	0.78	4.13
	50	1.04	10.21
	100	0.76	21.70
PP2	5	0.5	0.90
	10	1.2	1.99
	20	1.67	4.24
	50	0.94	10.52
	100	0.27	21.12

## Data Availability

The original contributions presented in this study are included in the article. Further inquiries can be directed to the corresponding author.
